# Predicting osteoporosis with body compositions in postmenopausal women: a non-invasive method

**DOI:** 10.1186/s13018-021-02351-3

**Published:** 2021-03-24

**Authors:** Wei-Hsiu Hsu, Wei-Bin Hsu, Chun-Hao Fan, Robert Wen-Wei Hsu

**Affiliations:** 1 Department of Orthopaedic Surgery, Chang Gung Memorial Hospital, No. 6, West Section, Chia-Pu Road, Pu-Tz City, Chiayi 613 Taiwan; 2 School of Medicine, Chang Gung University, Taoyuan City, Taiwan; 3 Sports Medicine Center, Chang Gung Memorial Hospital, No. 6, West Section, Chia-Pu Road, Pu-Tz City, Chiayi 613 Taiwan

**Keywords:** Basal metabolic rate, Fat-free mass, Fat mass, Osteoporosis, Postmenopausal women

## Abstract

**Background:**

The prevalence of osteoporosis is rising steadily as the aging population increases. Bone mineral density (BMD) assessment is a golden standard to establish the diagnosis of osteoporosis. However, the accessibility and radiation exposure limited its role in community screening. A more convenient approach for screening is suggested.

**Methods:**

A total of 363 postmenopausal women over the age of 50 were included in this study and assessed with the body composition [including fat-free mass (FFM), fat mass (FM), and basal metabolic rate (BMR)] and BMD. Normal distributions and correlation coefficients among variables were calculated using the Shapiro-Wilk test and Pearson’s correlation analysis, respectively. A receiver operating characteristic (ROC) curve was plotted, and the area under ROC curves (AUC) was determined to obtain the optimal cutoff values of the body composition variables for osteoporosis prediction.

**Results:**

The correlation coefficient of FFM, FM, FM ratio, and BMR with femur neck *T*-score was 0.373, 0.266, 0.165, and 0.369, respectively, while with spine *T*-score was 0.350, 0.251, 0.166, and 0.352, respectively (*p* < 0.01 for all). FFM, FM, and BMR showed an optimal cutoff value of 37.9 kg, 18.6 kg, and 1187.5 kcal, respectively, for detecting osteoporosis.

**Conclusions:**

The present study provided a model to predict osteoporosis in postmenopausal women, and the optimal cutoff value of FFM, FM, and BMR could be calculated in the Asian population. Among these factors, BMR seemed a better predictor than others. The BMR could be a target for exercise intervention in postmenopausal women for maintaining or improving BMD.

**Trial registration:**

ClinicalTrials.gov, NCT02936336. Retrospectively registered on13 October 2016.

**Supplementary Information:**

The online version contains supplementary material available at 10.1186/s13018-021-02351-3.

## Background

Osteoporosis is a common and silent skeletal disease characterized by bone mineral density (BMD) loss, resulting in fragile bone and high fracture risk [1–3]. It is more prevalent in postmenopausal women as a result of estrogen deficiency. Indeed, the prevalence of osteoporosis is rising steadily resulting from the aging population [4]. However, osteoporosis also occurs in healthy premenopausal women, resulting from various factors such as unsuitable diet, inadequate physical activity, medications, and smoking [5, 6]. In 2016, it was estimated that 200 million people suffered from osteoporosis, and 8.9 million had osteoporotic fractures [7]. Osteoporotic fracture has a high mortality and morbidity, increased cost of social care, and low health-related quality of life [1, 3, 8, 9]. Early detection and interventions such as supplemental calcium and vitamin D intake, bisphosphonates, and monoclonal antibody medications, and exercise (jogging, aerobic dancing, etc.) are suggested in accordance with disease severity for geriatric welfare [10]. According to the World Health Organization (WHO) guideline, osteoporosis diagnostic criteria and categories are based on BMD measurement. In general, dual-energy X-ray absorptiometry (DXA) is a hospital-based examination to estimate the BMD, while disadvantaged by its radiation exposure and limited accessibility in the community. Although various questionnaire tools for screening have been developed, they are limited by inadequate specificity. Bioelectrical impedance analysis (BIA) is a commonly used method for estimating body composition. Therefore, it is possible to improve osteoporosis detection through BIA because of its accessibility in the community.

Fat mass (FM) has a positive correlation with BMD, especially in the elderly [11–13], and is more influential on the BMD than fat-free mass (FFM) be [14, 15]. Some researchers pointed out that FFM is an important predictor for BMD without age restriction [16, 17]. Our previous study found out the basal metabolic rate (BMR) is closely associated with BMD in elderly persons and claimed BMR might be a predictor for osteoporosis [18]. Based on these results, the relationship between body composition and BMD was clarified, and the parameters of body composition (FFM, FM, FM ratio, and BMR) have the potential to predict BMD. But it is unclear what the cutoff values of FFM, FM, FM ratio, and BMR are for osteoporosis prediction. The prevalence of osteoporosis in women is higher than that in men [19]. Here, we undertook this pilot study to determine the cutoff values of FFM, FM, BMR, and FM ratio from body composition, non-invasive assessment, to predict osteoporosis.

## Methods

### Participants

This cross-sectional study was performed at the Chia-Yi Chang Gung Memorial Hospital. All participants were assessed with the BMD measurement and body composition. Based on the WHO osteoporosis definition, the subjects whose *T*-score > −2.5 were divided into the non-osteoporosis group while *T*-score < −2.5 into the osteoporosis group. All participants were enrolled from the rural community from southern Taiwan between August 2010 and December 2012. The inclusion criteria were physically independent postmenopausal women aged over 50 years old. The exclusion criteria were women taking any medications predisposing to poor bone quality, undergoing any medical therapies of osteoporosis and hormone-replacement therapy, with cognitive impairment and diabetes mellitus, and having bone fracture history. This study was approved by the Ethics Committee and Institutional Review Board of the Chang Gung Memorial Hospital (IRB 99-3951B) and registered in the ClinicalTrails.gov (ID: NCT02936336). The written informed consent was obtained from all individual participants included in the study.

### BMD measurement

**Fig. 1:**
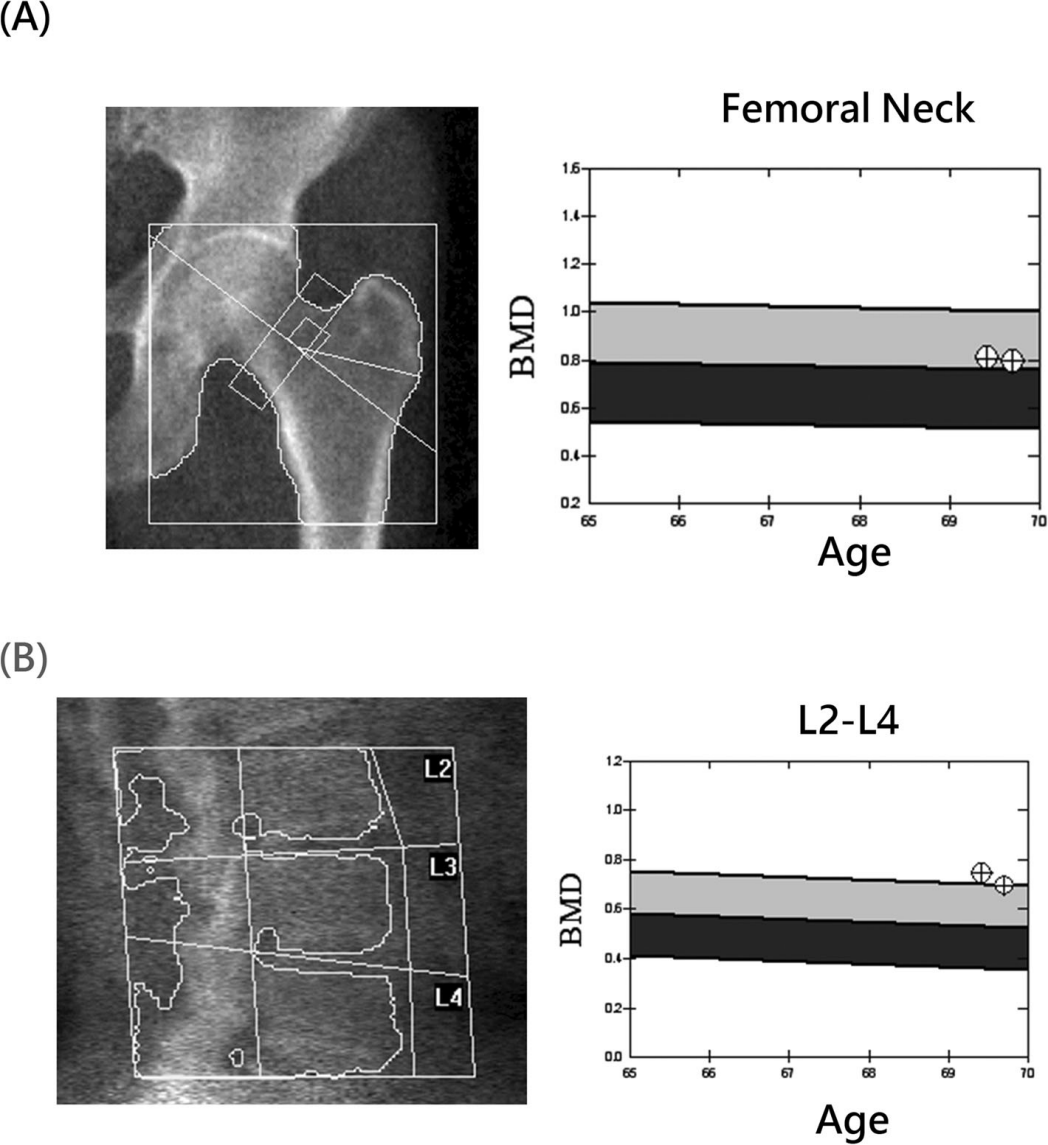
The example of BMD measurement of the **a** femoral neck and **b** lumbar spine L2–L4 by DXA in female aged 69

DXA (QDR 4500A; Hologic, Waltham, MA, USA) was performed to measure the BMD of the proximal femur (femoral neck) and lumbar spine (L2-L4) by an experienced and qualified radiographer (Fig. 1). By the definition from the World Health Organization, a BMD *T*-score of −2.5 or below is diagnosed as osteoporosis.

### Body composition

An eight-polar tactile-electrode impedance meter (InBody 720, Biospace, Seoul, Korea) was used to assess the body composition and simultaneously recorded body weight, FFM, total body water, regional fat mass, and BMR [18].

### Statistical analysis

Normal distributions were calculated using the Shapiro-Wilk test. Pearson’s correlation analysis was used to calculate the correlation coefficients among variables. By the World Health Organization definition (osteoporosis: *T*-score ≦−2.5; osteopenia: *T*-score ≦−-1 and >−2.5; normal: *T*-score >−1), the participants in this study were divided into two groups, non-osteoporosis (non-OP) and osteoporosis (OP). A receiver operating characteristic (ROC) curve was plotted with non-OP as positive and OP as negative to assess the diagnostic value of the predictors FFM, FM, BMR, and FM ratio. The area under the ROC curves (AUC) was calculated to evaluate the predictive performance of the variables. The points on the fit curve closest to the left upper corner were defined as cutoff points for the diagnosis of osteoporosis. All data analysis was performed using the Statistical Package for the Social Sciences Windows, version 17.0 (SPSS, Chicago, IL, USA). All continuous data were presented as the mean ± SD. The statistical analysis was performed by CHF.

## Results

Between August 2010 and December 2012, all participants were enrolled from the rural community from southern Taiwan. A total of 363 postmenopausal women over the age of 50 were included in this study. Based on the WHO osteoporosis definition, 211 subjects whose *T*-score > −2.5 were classified as non-osteoporosis group and 152 subjects whose *T*-score < −2.5 as osteoporosis group. The subjects’ demography is shown in Table 1. The average subjects’ age is 64.1 years, and body mass index (BMI) is 24.7 kg /m^2^. The women had a mean *T*-score of the femur neck and spine of respectively −1.9 ± 1.0 and −1.8 ± 1.4. Their mean FFM, FM, and FM ratio are respectively 38 ± 4 kg, 21.2 ± 6.3 kg, and 35 ± 7%, while the mean of BMR is 1182.7 ± 88.9 kcal. Age, height, weight, BMI, femur neck *T*-score (FNTS), spine *T*-score (STS), FFM, FF, and BMR had significant differences between the two groups. So, we assessed the relationship between BMD *T*-score (femur neck and spine) and other variables by Pearson correlation analysis. As shown in Table 2, age is negatively correlated with FNTS and STS while other variables are positively correlated with FNTS and STS (*p* < 0.01 for all). The correlation coefficient of FFM, FM, FM ratio, and BMR with femur neck *T*-score was 0.373, 0.266, 0.165, and 0.369, respectively, while with spine *T*-score 0.350, 0.251, 0.166, and 0.352, respectively. Meanwhile, we performed a multivariate logistic regression analysis of osteoporosis that showed age, FM ratio, and BMR were significant factors (Supplemental Table 1).

**Table 1:** Demographics for subjects (*N* = 363)

	Total subjects (*N* = 363)		Non-osteoporosis (*N* = 211)		Osteoporosis (*N* = 152)	
	Mean	±SD	Mean	±SD	Mean	±SD
Age (years)	64.1	±8.3	62.0	±8.0	66.9	±7.9*
Height (cm)	154.7	±5.2	155.5	±5.3	153.5	±4.7*
Weight (kg)	59.0	±8.5	61.0	±8.4	56.3	±8.0*
BMI	24.7	±3.4	25.2	±3.4	24.0	±3.3*
FNTS	−1.9	±1.0	−1.3	±0.7	−2.7	±0.7*
STS	−1.8	±1.4	−1.0	±1.1	−2.8	±1.1*
FM ratio (%)	35.4	±6.7	35.9	±6.4	34.7	±7.1
FFM (kg)	37.6	±4.1	38.8	±4.2	36.0	±3.4*
FF (kg)	21.2	±6.3	22.2	±6.2	19.9	±6.3*
BMR (kcal)	1182.7	±88.9	1207.3	±90.7	1148.5	±73.9 *

*BMR* basal metabolic rate, *FFM* fat-free mass, *FM* fat mass, *FM ratio* fat mass ratio, *FNTS* femur neck *T*-score, *STS* spine *T*-score

*p ≤ 0.05 between non-osteoporosis and osteoporosis

**Table 2:** Correlation coefficients between variables and BMD *T*-score (*r*)

	FNTS	STS	Age	Height	Weight	BMI	FM ratio	FM	BMR	FFM
FNTS	1									
STS	.619**	1								
Age	−.353**	−.222**	1							
Height	.200**	.180**	−.206**	1						
Weight	.356**	.345**	−.064	.348**	1					
BMI	.255**	.265**	.029	−.123*	.874**	1				
FM ratio	.165**	.166**	.069	−.205**	.646**	.773**	1			
FM	.266**	.251**	.038	.001	.859**	.894**	.908**	1		
BMR	.369**	.352**	−.151**	.627**	.662**	.387**	−.066	.281**	1	
FFM	.373**	.350**	−.160**	.621**	.665**	.393**	−.054	.284**	.995**	1

*BMR* basal metabolic rate, *FFM* fat-free mass, *FM* fat mass, *FM ratio* fat mass ratio, *FNTS* femur neck *T*-score, *STS* spine *T*-score

*p ≤ 0.05

**p ≤ 0.01

**Fig. 2:**
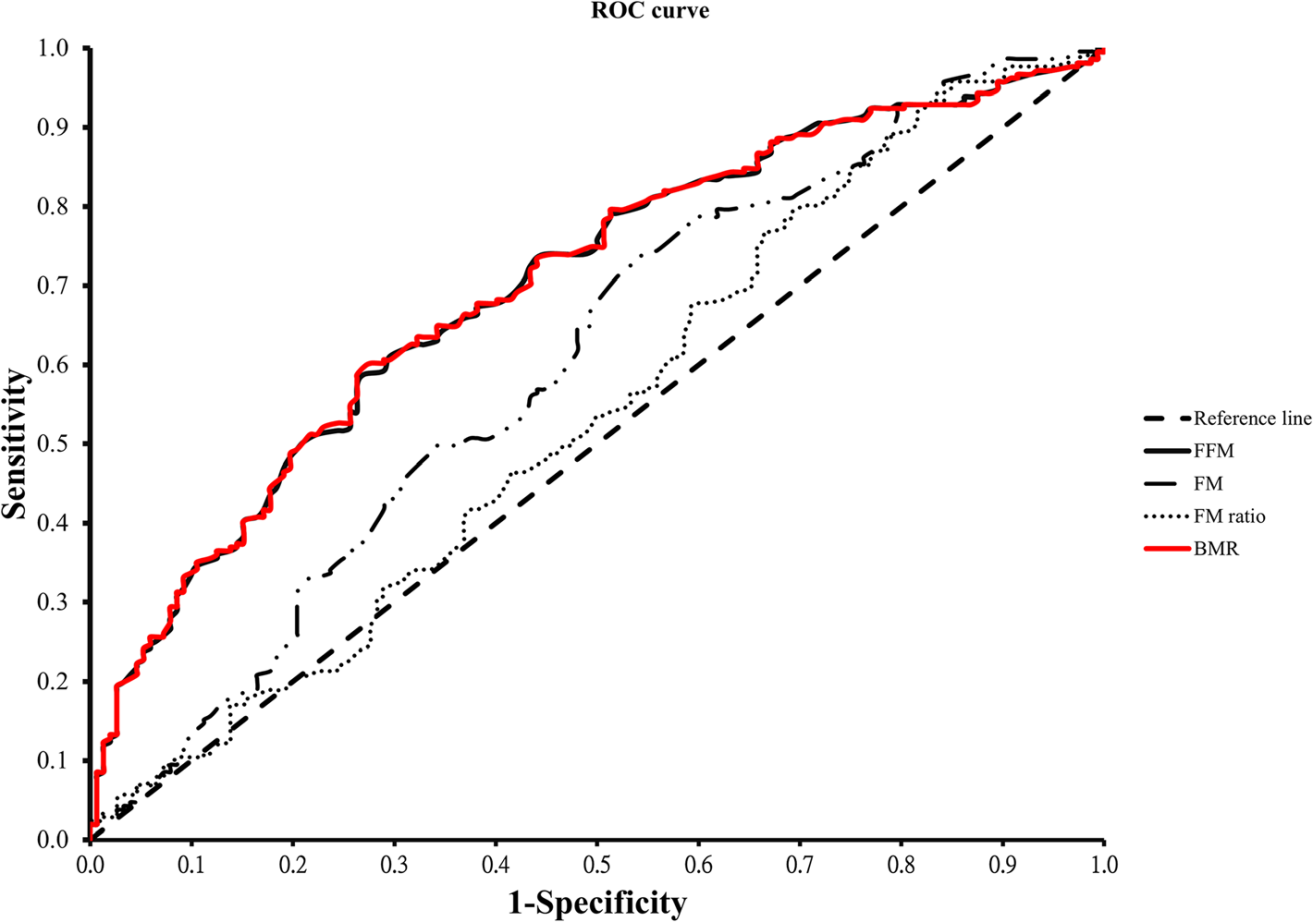
ROC analysis to identify the optimal cutoff value of FFM, FM, FM ratio, and BMR to osteoporosis. Relationship between sensitivity and specificity at different cutoff scores in various variables

ROC curve analysis was performed to estimate the optimal cutoff value of FFM, FM, FM ratio, and BMR for predicting osteoporosis. As shown in Table 3 and Fig. 2, FFM showed an optimal sensitivity (0.588) and specificity (0.73) at the cutoff value of 37.9 kg (AUC = 0.700, 95%CI 0.646–0.753). FM revealed an optimal sensitivity (0.73) and specificity (0.47) at the cutoff value of 18.6 kg (AUC = 0.602, 95%CI 0.542–0.661). BMR presented an optimal sensitivity (0.59) and specificity (0.73) at the cutoff value of 1187.5 kcal (AUC = 0.701, 95%CI 0.647–0.754) while FM ratio did not statistically predict osteoporosis.

**Table 3:** Area under the curve (AUC) of BMR, fat-free mass, and fat mass as a single predictor for each measurement site from ROC analysis

Variable	AUC	Cut-point	1-specificity	Sensitivity	95.0% CI	
					Lower	Upper
FFM	.700**	37.9 kg	.270	.588	.646	.753
FM	.602**	18.6 kg	.533	.730	.542	.661
FM ratio	.538	26.7%	.829	.934	.477	.599
BMR	.701**	1187.5 kcal	.270	.597	.647	.754

*FFM* fat-free mass, *FM* fat mass, *FM ratio* fat mass ratio, *BMR* basal metabolic rate

**p < 0.01

## Discussion

Previous studies reported that FM is related to the BMD of the lumbar spine and proximal femur [20, 21]. Besides, other studies showed that BMR is more closely related to BMD than FFM, FM, and BMI [18, 22, 23]. Our results were echoed to these studies and revealed that BMR could be effective to predict osteoporosis (BMD *T*-score < −2.5) with the optimal cutoff value of 1187.5 kcal (Table 3 and Fig. 2) in women over 50 years old. If their BMR was lower than the cutoff values 1187.5 kcal, they might have a high risk of osteoporosis.

As mentioned above, higher FM went along with higher BMD as an effective predator for osteoporosis, but some studies showed that FM was not effective for osteoporosis prediction in middle-aged and elderly people in Asia [24, 25], and the increased central body FM was negatively associated with BMD [26]. Moreover, Saarelainen and his colleagues reported that trunk FM is positively related to lumbar spine BMD, but not to the hip BMD and body weight in postmenopausal women [21]. Kirchengast and his colleagues found out that the relationship between FM and BMD only occurred in elder women [27]. These studies showed that the FM for osteoporosis prediction might be restricted by ethnicity, gender, the region of FM, etc. However, FFM has a stronger positive relation with BMD at all ages in Chinese men and women [28], and our results (Table 3) also showed that the AUC of FFM (0.700) was higher than FM (0.602), suggesting that FFM seems to be better than FM for osteoporosis prediction. However, BMD is influenced by multiple factors such as genetic factors, gender, diet physical activity, medical diseases, and stress [5, 29]. It seemed the BMR is not the sole determinant factor for osteoporosis that the correlation coefficient between all body composition variable and BMD *T*-score was < 0.4. However, it did provide a window for screening osteoporosis through a convenient and non-invasive methodology.

BMR, the amount of energy expended, is predicted with regard to resting energy expenditure. In multivariate analysis, BMR was low correlated with FM (correlation coefficient 0.281) (Table 2), suggesting that BMR and FM might be independent predictors of osteoporosis. However, BMR is more closely associated with BMD in elderly persons than BMI, FM, and FFM [18, 22, 23]. BMR is positively associated with muscle strength [30] while muscle strength and BMD also are correlated [31, 32]. Our results also showed that the AUC of BMR is higher than FFM and FM, suggesting that BMR might be a good predictor for osteoporosis. According to our results, we proposed that if the postmenopausal woman’s BMR is lower than the cutoff value,1187.5 kcal, the subject might have a higher osteoporosis risk than others with over 1187.5 kcal. Although hip and spine fractures are a portion of osteoporotic fractures, these fractures have a huge impact on the patient’s daily activity and medical burden [33, 34]. Low BMD is associated with an increased risk of fracture and hence provided a measurable method in osteoporotic fracture preventions. However, the disadvantages are radiation exposure and limited accessibility. Since BMR could well predict BMD in the present study, it seemed a good method in screening osteoporosis. Besides, the American College of Sports Medicine (ACSM) proposes that increasing physical exercise can maintain and improve bone quality in response to bone health problems [35]. Here, we provided the cutoff value of 1187.5 kcal of BMR. It could serve as a target value for exercise intervention to enhance BMR in postmenopausal women to maintain and improve their BMD.

Several limitations of the present study should be acknowledged. First, the subjects of this study are postmenopausal women aged over 50 years old. Men and those under 50 years old are not included. Therefore, the relevant threshold only applies to postmenopausal women aged over 50 years old. Second, because the subjects were from southern Taiwan, the present cutoff value was restricted to Asians. It was shown that the body compositions were not identical between Caucasian and Asian populations **[**36–38]. In fact, Asian populations had more fat mass percentage and central fat. Therefore, extrapolating the findings in the present study to Caucasian populations warranted further investigations. Nevertheless, the effect of the exercise on the BMD has no difference in different races, so we proposed that the BMR could be a predictor for BMD in different races via slightly adjusting the cutoff value of BMR.

## Implications for practice

### Conclusions

In the present study, our results showed that BMR was a better predictor for osteoporosis than other body compositions, including FM, FM ratio, and FFM. We proposed that the BMR could serve as a screening tool to alert the risk of osteoporosis and early intervention for osteoporosis. Simultaneously, the cutoff value of BMR also could be a target value for exercise intervention to enhance BMR in postmenopausal women to maintain or improve their BMD. In addition, clinical risk assessment instruments, such as Osteoporosis Self-Assessment Tool, the Simple Calculated Osteoporosis Risk Estimation instrument, the Osteoporosis Self-Assessment Tool for Asians, and the Osteoporosis Risk Assessment Instrument, all had high sensitivity exceeding 90% for identifying individuals with DXA-determined osteoporosis or low BMD but low specificity at thresholds required for high sensitivity [39]. It is possible to include this objective data such as BMR to enhance sensitivity and specificity in osteoporosis screening.

## Supplementary Information

**Additional file 1: Supplemental Table 1.** Multivariate logistic regression analysis for predicting osteoporosis.

## Data Availability

The datasets used and/or analyzed during the current study are available from the corresponding author on reasonable request.

## Abbreviations

BMD: Bone mineral density
FFM: Fat-free mass
FM: Fat mass
BMR: Basal metabolic rate
ROC: Receiver operating characteristic
AUC: Area under the ROC curves
BMI: Body mass index
ACSM: American College of Sports Medicine
