# Supplementary material for: Predicting osteoporosis with body compositions in postmenopausal women: a non-invasive method
Source: J Orthop Surg Res. 2021 Mar 24;16:215. doi: 10.1186/s13018-021-02351-3 (PMC7989015; doi:10.1186/s13018-021-02351-3)
Supplement: Supplementary file 1 — Additional file 1: Supplemental Table 1. Multivariate logistic regression analysis for predicting osteoporosis. [file 13018_2021_2351_MOESM1_ESM.docx]

**Supplemental Table 1. Multivariate logistic regression analysis for predicting osteoporosis**

|  | Odds ratio | 95% confidence interval | | *p* value |
| --- | --- | --- | --- | --- |
|  |  | Lower | Upper |  |
| Age | .929 | .902 | .957 | .000 ** |
| Height | .944 | .859 | 1.036 | .224 |
| BMI | .872 | .669 | 1.136 | .311 |
| FM ratio | 1.259 | 1.039 | 1.525 | .019 * |
| FM | .866 | .694 | 1.080 | .201 |
| BMR | 1.033 | 1.001 | 1.066 | .041 * |
| FFM | .710 | .384 | 1.313 | .275 |

BMI: Body mass index, BMR: Basal metabolic rate, FFM: Fat-free mass, FM: Fat mass, FM ratio: Fat mass ratio. * *p* ≤ 0.05. ** *p* ≤ 0.01
